# Assessing the Effect of Composting Cassava Peel Based Substrates on the Yield, Nutritional Quality, and Physical Characteristics of *Pleurotus ostreatus* (Jacq. ex Fr.) Kummer

**DOI:** 10.1155/2014/571520

**Published:** 2014-12-17

**Authors:** N. K. Kortei, V. P. Dzogbefia, M. Obodai

**Affiliations:** ^1^Department of Nuclear Agriculture and Radiation Processing, Graduate School of Nuclear and Allied Sciences, P.O. Box 80, Legon, Ghana; ^2^Department of Biochemistry and Biotechnology, Faculty of Biosciences, KNUST, PMB, Kumasi, Ghana; ^3^CSIR-Food Research Institute, P.O. Box M 20, Accra, Ghana

## Abstract

Cassava peel based substrate formulations as an alternative substrate were used to grow mushrooms. The effect of two compost heights, three composting periods on the mycelia growth, physical characteristics, yield, and nutritional qualities of* Pleurotus ostreatus* (Jacq. ex Fr.) Kummer was studied. Mean mycelia growth of 16.2 cm after a period of seven (7) weeks was the best for 1.5 m compost height. Cap diameter and stipe length differed significantly (*P* < 0.05) with the compost heights (0.8 m and 1.5 m). The yield on compost height of 1.5 m, composted for 5 days, differed significantly (*P* < 0.05) from that of 0.8 m and gave increasing yields as follows: cassava peels and manure, cassava peels only, cassava peels and corn cobs (1 : 1 ratio), and cassava peels and corn cobs (1 : 1 ratio) with chicken manure. Composting periods (3 and 7 days) gave varying yields depending on the compost height. Based on the findings an interaction of 1.5 m compost height and 5 days composting period on cassava peels and corncobs (1 : 1 ratio) with chicken manure produced the best results. The nutritional quality of the mushrooms also differed significantly (*P* < 0.05), indicating that cassava peels could be used as a possible substrate in cultivation of mushroom.

## 1. Introduction

In the cultivation of mushrooms, various lignocellulosic wastes are used as substrates and these act as sources of nutrients for their growth [1]. These wastes include, among other cereal grains, rice straw, wheat straw, cottonseed hulls, soybean meal, and sawdust [2–5]. Due to varying nutrients in the substrates, different mushroom yields have been recorded by various workers [6, 7].* Pleurotus *spp. are macrofungi which utilize polysaccharides (cellulose and hemicelluloses) from various lignocelluloses to produce expensive protein for human consumption [8, 9]. Their global economic value is now incredible, and the reason for the rise in consumption is a combination of their value as food [10, 11] and their medicinal or nutraceutical properties [12–15].

Composting is an aerobic process in which microorganisms convert a mixed organic substrate into carbon dioxide (CO_2_), water, minerals, and stabilized organic matter. It is a solid-state fermentation process, which exploits the phenomenon of microbial degradation and mineralization [16, 17]. Control of environmental conditions during the process distinguishes composting from natural rotting or decomposition [18]. Controlled conditions, particularly of moisture and aeration, are required to yield temperatures (49°C–60°C) conducive to the microorganisms involved in the composting process [19]. Temperature is the main factor that controls microbial activity during composting [20].

Cassava (*Manihot esculenta *Crantz, Euphorbiaceae) is the sixth most important food crop globally, in terms of annual production [21], and is a staple food for approximately 800 million people [22, 23]. This perennial root crop is grown in the tropics, including sub-Saharan Africa, Asia, Pacific Islands, and Central and South America [23–25]. In Ghana its annual production is approximately 6.6 million metric tons [26] and total production for 2010 was 13,504,100 tonnes [21]. The peel is a byproduct of processing the roots for starch, cassava flour, and “gari” (a fermented cassava meal product) which constitute 11% of the root, with approximately 400,000 MT (dry matter basis) of it produced annually [27]. Cassava peels and corncobs are lignocellulosic materials which consist of three main components, namely, cellulose, hemicellulose, and lignin [28, 29].

This paper presents the effect of composting height, period, and supplementation with chicken manure on the yield, nutritional quality, and some physical characteristics of* Pleurotus ostreatus* (Jacq. ex Fr.) Kummer cultivated on different cassava peel based substrate formulations.

## 2. Materials and Methods

### 2.1. Spawn and Compost Preparation

Cultures of* P. ostreatus* (Jacq. ex Fr.) Kummer strain EM-1 originally from Mauritius were maintained on potato dextrose agar slants and used to prepare sorghum grain spawn [33]. Compost was prepared by the outdoor single-phase solid waste fermentation. Dried cassava peels and corncobs substrates were reduced to average particle sizes of 0.5 cm^2^ and 1.2 cm^2^, respectively. Substrates were obtained from Kwame Nkrumah University of Science and Technology (KNUST) campus and its environs, Kumasi. The substrates were mixed with chicken manure and lime and composted as described by [34]. The mixture was then stacked into a heap of 1.5 m height and 1.5 m base width, as well as 0.8 m height and 0.8 m width, and left to compost for varying number of days (3, 5, and 7) with regular turnings every 2 days. Moisture content was adjusted to approximately 68–70% [35]. Compost sizes (105 kg and 315 kg) used in this study were directly related to the respective compost heights (0.8 m and 1.5 m).

### 2.2. Preparation of Substrate Mixtures

At the end of the composting period, varying combinations of cassava peels and corncob mixtures were prepared and bagged. Substrate mixtures obtained were as shown in Table 1.

### 2.3. Bagging

One kilogram of each substrate mixture was bagged into heat resistant polypropylene bags of dimension 29 × 9 cm. For each treatment, 8 replicates were used.

### 2.4. Sterilization

The bagged substrates were then sterilized with moist heat in drums at temperatures of 95–100°C for 2.5 hours.

### 2.5. Inoculation, Incubation, and Cropping

The bags were inoculated with 5 g of spawn and incubated at ambient temperature (28–32°C) for approximately 35 days. From the incubation room, the bags were sent to the cropping house where the compost bags were placed on horizontal shelves. They were slit open at the neck in the cropping house where humidity of 80–85% was maintained by watering twice a day.

### 2.6. Mycelial Growth, Cap Diameter, Stipe Length, and Yield Measurements

- Mycelial growth = (longest growth + shortest growth)/2
- Average cap diameter = (longest + shortest cap diameter)/2
- Stipe length = length of cap base to end of stalk
- Yield = biological efficiency (B.E) (%) = fresh weight of mushrooms/dry weight of substrates × 100.

### 2.7. Proximate Analysis

All the moisture, fat, ash, protein, and carbohydrate content were determined by Association of Official Analytical Chemists Methodology [36].

### 2.8. Dietary Fibre

The content of soluble, insoluble, and total fibre was determined using AOAC 991.43 method [36].

### 2.9. Statistical Analysis

All experiments performed were subjected to analyses of variance (one-way ANOVA) and then significant differences were determined using Duncan's multiple range test (DMRT) with SPSS 16 (Chicago, USA).

## 3. Results and Discussion

### 3.1. Mycelial Growth

**Figure 1:**
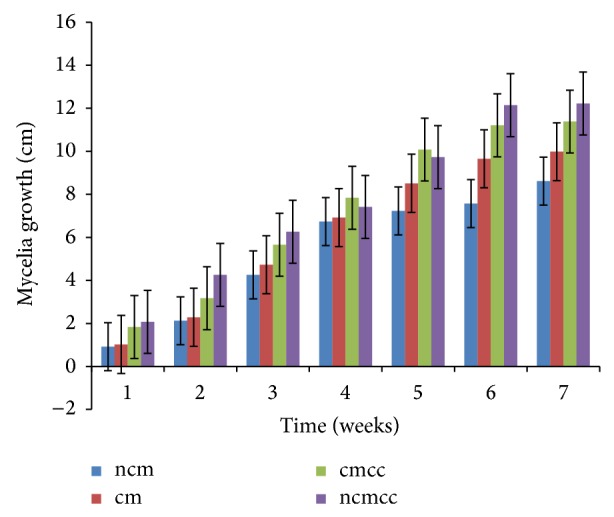
Weekly mycelial growth on substrates of 0.8 m compost height and 3 days' composting period.

**Figure 2:**
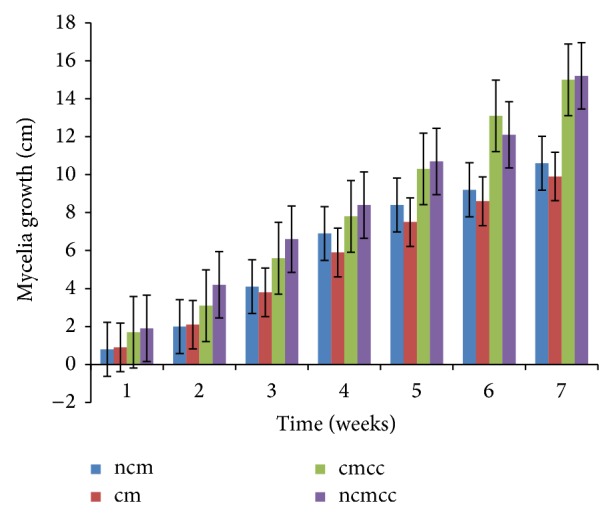
Weekly mycelial growth on substrates of 0.8 m compost height and 5 days' composting period.

**Figure 3:**
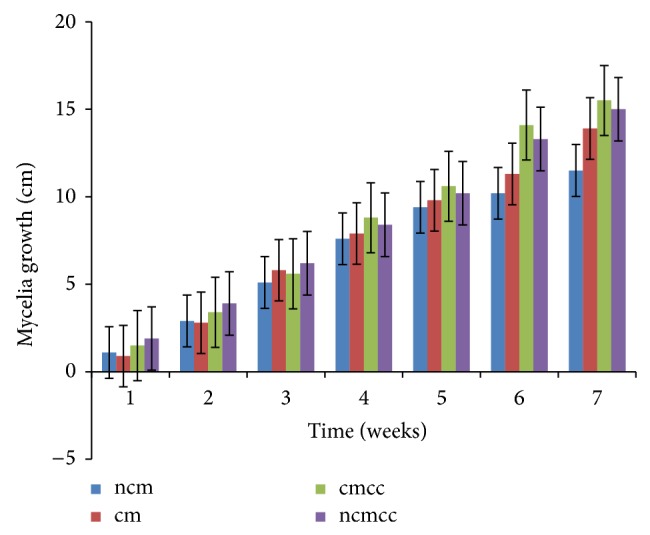
Weekly mycelial growth on substrates of 0.8 m compost height and 7 days' composting period.

**Figure 4:**
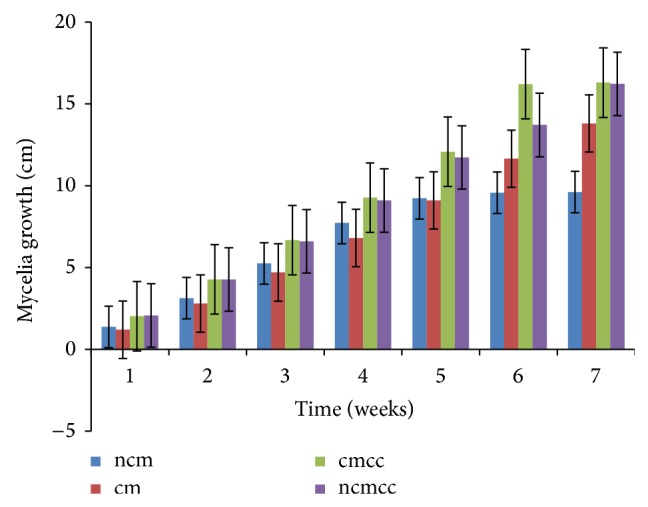
Weekly mycelial growth on substrates of 1.5 m compost height and 3 days' composting period.

**Figure 5:**
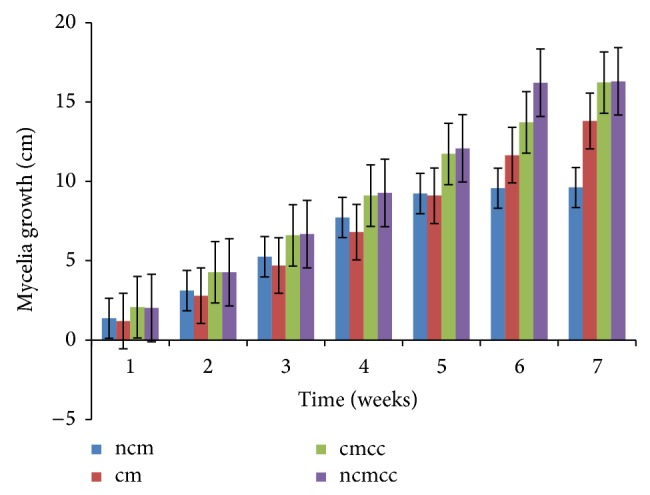
Weekly mycelial growth on substrates of 1.5 m compost height and 5 days' composting period.

**Figure 6:**
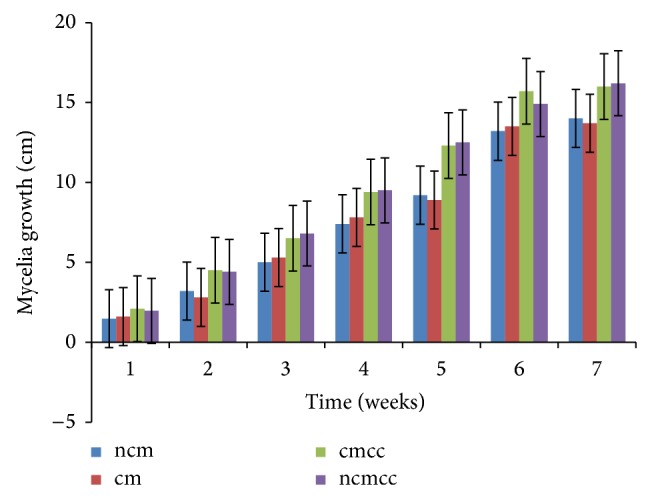
Weekly mycelial growth on substrates of 1.5 m compost height and 7 days' composting period.

The various substrate combinations and composting treatments (Table 1) resulted in different growth responses due to the relative distribution of nutrients. Mycelial growth was significantly (*P* < 0.05) affected by compost height, composting period, substrate combination, and supplementation (Figures 1, 2, 3, 4, 5, and 6). Higher compost heights and longer composting periods provided sufficient temperatures for microbial activities which allowed for greater decomposition of polysaccharides into smaller units for usage by microorganisms and mushroom mycelia. This was evident in producing the longest mycelia length of 16.3 cm on ncmcc (50% cassava peels + 50% corncobs + no chicken manure) of 5 days' composting period and 1.5 m height at the end of the seventh (7th) week of incubation (Figure 5). The shortest mycelia length of 8.1 cm was recorded by 100% cassava peels composted for 3 days of 0.8 m height (Figure 1). Generally, cm and ncm (100% cassava peels + chicken manure and 100% cassava peels + no chicken manure, resp.) and their interactions performed poorly. Conversely, mixture of cassava peels and corncobs (1 : 1 ratio) and its interactions supported good mycelia growth perhaps because of its porosity and high proportion of cellulose [37]. Mixtures of various agricultural wastes have been reported by Akinyele and Adetuyi [38] to give good yields of mushroom mycelia. Additionally, this substrate mixture possesses a better C/N ratio of about 159.12 compared to the C/N ratio of cassava peels (Table 2) [28]. This agrees with the findings of Mantovani et al. [39] who reported that greater C/N ratios promoted good fungal growth as they investigated the effect of the addition of nitrogen sources to cassava fiber and carbon-to-nitrogen ratios on fungal growth.

Interplay of these factors on mycelia colonization rate confirms the fact that both the substrate formula and the strain used affect mycelial growth rate and therefore the incubation and crop cycle duration [2, 39]. There was no significant (*P* > 0.05) difference in the growth of mycelia on combined substrates of 50% cassava peels and 50% corncobs whether supplemented or not.

### 3.2. Yield

The yield of mushrooms obtained from the two compost heights (0.8 m and 1.5 m) studied differed significantly (*P* < 0.05). Comparing the yield of mushrooms harvested from the two compost heights, higher yields were generally obtained from compost height 1.5 m and its interactions of composting periods the various substrates (Table 3(a)) than the 0.8 m compost height and its interactions (Table 3(b)). This trend suggests that the period of composting and the height for composting were insufficient to support microbial activity [20]. Also, the degree of decomposition in the compost heights (0.8 m and 1.5 m) with respect to nutrients available may account for the differences in the yield of mushrooms harvested [33].

An interaction of 1.5 m compost height 5 days' composting period and a substrate mixture of cassava peels and corncobs (1 : 1 ratio) supplemented with chicken manure produced the highest yield of 299 g (Table 3(b)) and the lowest yield of 163 g (Table 3(a)) from ncm (100% cassava peels + no chicken manure), 0.8 m and 5 days composting period. Essentially, previous works done by Hudson [40] and Carlile and Watkinson [41] suggest that nutrient release during aerobic fermentation of composting is temperature dependent so higher compost heights (larger size) will result in higher temperatures, which, according to Vetayasuporn [42], support effective microorganisms to digest (mainly by lignocellulolytic enzymes) the substrate and subsequently release different sugars. Digestion of cellulose produces glucose and cellobiose, while digestion of hemicellulose produces mostly xylose and other sugars, such as galactose, mannose, arabinose, pyranose, glucuronic acid, and galacturonic acid as secondary products [43–45]. These are converted into sources of carbon which are easily utilized for growth of mycelia, primordial initiation, fruit body formation, and ultimately higher yields. There was a general inverse correlation of yield to flush number increase (Tables 3(a) and 3(b)). This could be attributed to depletion of nutrients in the substrate and accumulation of some metabolites which inhibited growth [1]. Yields obtained in this study were lower than results reported by some researchers [35, 46]. However yields were within range of results reported by [47].

### 3.3. Physical Characteristics

The cap diameter and stipe lengths of* P. ostreatus* grown on different substrate mixtures differed significantly (*P* < 0.05) as a result of probable presence of little or complete lack of some vital nutrients, especially nitrogen, needed for* P. ostreatus* growth in cassava peel. Comparatively smaller sizes were recorded for 0.8 m compost height, composting period, and substrate interactions. The ranges of cap diameter and stipe lengths were 6.5–3.6 cm and 5.5–1.8 cm, respectively (Table 10).

On the other hand, bigger sizes were recorded for 1.5 m compost height, composting periods, and substrate mixture interactions. They were in the ranges 7–5 cm and 5.2–4.1 cm for cap diameter and stipe lengths, respectively (Table 10). Nitrogen and carbon are two essential elements required for cellular functions for growth and various metabolic activities, particularly protein and enzymes synthesis [43]. Carbon is readily available from cellulose, hemicellulose, and lignin from the substrates, but nitrogen occurs mainly in a bound form and is not available until it is enzymatically released. Absence or limited supply of any of these elements may result in poor physical growth.

Raymond et al. [47] reported that the yield of* Pleurotus* mushroom could be boosted by the addition of nitrogenous supplements. The ranges were in agreement with [11, 48–50]. Analysis of cap diameters and stipe lengths revealed significant differences (*P* < 0.05) between the two compost heights.

### 3.4. Nutritional Content

The various interactions of compost heights, composting periods, substrates, and environmental conditions resulted in significantly different (*P* < 0.05) nutritional compositions of mushrooms from this experiment.

The moisture content of the samples ranged from 83.3 to 85.6% (Table 4) within the category of high moisture foods, thus making them highly perishable [51]. High moisture contents promote susceptibility to microbial growth and enzyme activity [51]. Works of researchers [52, 53] reported comparable values of 84-85%.

The ash content ranged from 7.32 to 7.83% (Table 5). The differences in ash content for respective samples grown on different substrate formulations were not significant (*P* > 0.05). Ash content of foods represents their mineral element composition. Mushrooms are good bioaccumulators of mineral elements and that is evident in their medicinal attributes. Some mineral elements are needed in the body for the formation of red blood cells, formation of strong teeth and bones, and so forth [54].

These values were slightly higher than works of [54, 55] but were however lower than work of Aida et al. [56]. There were appreciable quantities of fiber in the mushroom samples examined. As shown in Table 6, the fibre content of the mushrooms ranged from 8.39 to 8.88%. This observation agrees with works of researchers [10, 57] who recorded similar values. There were no significant differences (*P* > 0.05) between the values obtained for mushrooms cultivated under the various conditions. Fungi derived *β*-glucans are notable for their ability to modulate the immune system [10, 58]. The values obtained under various growing conditions for fat were in the range of 2.14–2.22% (Table 7). There was no significant difference (*P* > 0.05). This range of fat content is lower than that of earlier report of Jaworska et al. [59] and much depends on the nature of substrate. However, lower values were obtained by Aida et al. [56].

Protein contents ranged from 10.48 to 10.80% (Table 8). They differed significantly (*P* < 0.05) with regard to the various interactions. Jaworska et al. [59] reported that not only the protein content of the substrate but also nature of protein in the substrate influences the protein content of the fruiting bodies. Values obtained were within range of works [52, 56] but lower than works of [10, 55, 57, 60, 61]. The carbohydrate values fell within the range of 73.3–74.5% (Table 9). They differed significantly (*P* < 0.05). The values obtained were higher than previous works [31, 32, 52] which recorded 65.8–66.8% values for total carbohydrate content in different* Pleurotus* species.

## 4. Conclusion

This study found out that compost size is directly related to the degree of decomposition which in turn accounts for nutrient release for growth of mushrooms. The overall best yield (299 g) was produced by the interaction of 1.5 m compost height, 5 days' composting period, and substrate mixture of cassava peels and corncobs (1 : 1 ratio) supplemented with chicken manure. It can therefore be concluded that greater compost heights give optimum yields and good physical attributes and nutrient quality.

## Figures and Tables

**Table 1:** Substrate compositions and codes of the experiment.

Substrate code	Substrate composition
cm	100% cassava peels + chicken manure
ncm	100% cassava peels + no chicken manure
cmcc	50% cassava peels + 50% corncobs + chicken manure
ncmcc	50% cassava peels + 50% corncobs + no chicken manure

Amount of chicken manure added was 16.5 kg (10% w/w).

**Table 2:** Chemical analysis of substrates (dry weight) used in experiments.

Waste	Moisture (%)	Ash (%)	Nitrogen (%)	Protein (%)	Carbon (%)	C/N	pH
Cassava peels	2.42	6.46	1	6.25	44.82	44.82	5.7
Corncobs	2.39	1.98	0.4	2.5	45.66	114.2	4.74
Chicken manure	3.3	18.50	2	30	7	3.5	6.3

Source: [28, 30–32].

**Table:** (a) −0.8 m compost height

Period (days)	Substrate	Flush					Total	B.E (%)
		1st	2nd	3rd	4th	5th		
3	cm	57	42	33	33	24	189^a^	27
	ncm	49	46	38	30	19	182^a^	26
	cmcc	59	50	45	34	31	219^b^	31.3
	ncmcc	56	55	50	29	28	218^b^	31.1

5	cm	59	51	49	40	29	228^c^	32.6
	ncm	45	42	31	27	23	168^a^	24
	cmcc	61	55	52	33	31	232^c^	33
	ncmcc	60	58	52	37	25	232^c^	33

7	cm	62	57	52	41	28	240^c^	34.3
	ncm	53	39	39	32	20	183^a^	26.1
	cmcc	64	60	57	38	22	241^c^	34.4
	ncmcc	56	58	54	43	25	236^c^	33.7

Means with the same letters in a column are not significantly different (*P* > 0.05). Results are mean scores of 8.

**Table:** (b) −1.5 m compost height

Period (days)	Substrate	Flush					Total	B.E (%)
		1st	2nd	3rd	4th	5th		
3	cm	48	46	45	37	19	195^a^	27.9
	ncm	50	45	45	32	30	202^a^	28.8
	cmcc	65	62	53	44	37	261^b^	37.3
	ncmcc	66	59	57	51	24	257^b^	36.7

5	cm	59	51	35	31	26	202^a^	28.9
	ncm	70	70	42	63	34	279^c^	39.9
	cmcc	71	64	58	52	43	288^c^	41.1
	ncmcc	71	72	59	50	45	297^d^	42.4

7	cm	63	53	40	37	30	223^a^	31.9
	ncm	68	60	55	53	39	275^c^	39.3
	cmcc	74	64	60	58	43	299^d^	42.7
	ncmcc	71	67	57	49	44	288^d^	41.1

Means with the same letters in a column are not significantly different (*P* > 0.05). Results are mean scores of 8.

**Table 4:** Average moisture content (%) of mushrooms harvested on different substrates.

Substrate type	0.8 M			1.5 M		
	Composting period			Composting period		
	3 days	5 days	7 days	3 days	5 days	7 days
CM	84.63 ± 0.01	84.57 ± 0.02	84.51 ± 0.02	85.48 ± 0.01	84.27 ± 0.03	84.11 ± 0.02
NCM	84.78 ± 0.03	84.83 ± 0.02	84.58 ± 0.01	84.63 ± 0.02	84.71 ± 0.01	84.63 ± 0.01
NCMCC	83.64 ± 0.02	84.39 ± 0.01	83.72 ± 0.01	84.37 ± 0.02	83.42 ± 0.02	83.33 ± 0.02
CMCC	84.80 ± 0.01	85.72 ± 0.02	84.58 ± 0.03	83.44 ± 0.01	84.51 ± 0.02	85.60 ± 0.03

Results are mean scores of 3 ± SE.

**Table 5:** Average ash content (%) of mushrooms harvested on different substrates.

Substrate type	0.8 M			1.5 M		
	Composting period			Composting period		
	3 days	5 days	7 days	3 days	5 days	7 days
cm	7.61 ± 0.1	7.83 ± 0.1	7.81 ± 0.3	7.48 ± 0.2	7.59 ± 0.2	7.64 ± 0.1
ncm	7.49 ± 0.2	7.47 ± 0.2	7.52 ± 0.2	7.61 ± 0.1	7.71 ± 0.2	7.69 ± 0.2
ncmcc	7.32 ± 0.1	7.41 ± 0.1	7.65 ± 0.3	7.70 ± 0.1	7.63 ± 0.3	7.82 ± 0.2
cmcc	7.55 ± 0.1	7.49 ± 0.3	7.60 ± 0.1	7.67 ± 0.2	7.34 ± 0.1	7.65 ± 0.1

Results are mean scores of 3 ± SE.

**Table 6:** Average fibre content (%) of mushrooms harvested on different substrates.

Substrate type	Compost height					
	0.8 M			1.5 M		
	Composting period			Composting period		
	3 days	5 days	7 days	3 days	5 days	7 days
cm	8.42 ± 0.14	8.47 ± 014	8.39 ± 0.13	8.72 ± 0.14	8.48 ± 0.14	8.69 ± 0.14
ncm	8.47 ± 0.13	8.69 ± 0.13	8.57 ± 0.15	8.68 ± 0.14	8.75 ± 0.14	8.88 ± 0.15
ncmcc	8.56 ± 0.15	8.77 ± 0.14	8.79 ± 0.14	8.80 ± 0.13	8.78 ± 0.14	8.74 ± 0.13
cmcc	8.73 ± 0.14	8.74 ± 0.14	8.86 ± 0.15	8.80 ± 0.13	8.81 ± 0.13	8.79 ± 0.14

Results are mean scores of 3 ± SE.

**Table 7:** Average fat content (%) of mushrooms harvested on different substrates.

Substrate type	Compost height					
	0.8 M			1.5 M		
	Composting period			Composting period		
	3 days	5 days	7 days	3 days	5 days	7 days
CM	2.16 ± 0.13	2.17 ± 0.14	2.17 ± 0.12	2.15 ± 0.13	2.16 ± 0.14	2.16 ± 0.14
NCM	2.14 ± 0.13	2.18 ± 0.13	2.20 ± 0.11	2.18 ± 0.12	2.17 ± 0.13	2.18 ± 0.13
NCMCC	2.17 ± 0.14	2.17 ± 0.13	2.16 ± 0.13	2.17 ± 0.14	2.19 ± 0.13	2.16 ± 0.14
CMCC	2.24 ± 0.12	2.19 ± 0.14	2.22 ± 0.12	2.18 ± 0.14	2.18 ± 0.14	2.15 ± 0.13

Results are mean scores of 3 ± SE.

**Table 8:** Average protein content (%) of mushrooms harvested on different substrates.

Substrate type	Compost height					
	0.8 M			1.5 M		
	Composting period			Composting period		
	3 days	5 days	7 days	3 days	5 days	7 days
CM	10.65 ± 0.13	10.60 ± 0.14	10.72 ± 0.12	10.62 ± 0.13	10.53 ± 0.14	10.69 ± 0.14
NCM	10.58 ± 0.13	10.54 ± 0.13	10.61 ± 0.14	10.68 ± 0.14	10.71 ± 0.12	10.48 ± 0.14
NCMCC	10.73 ± 0.12	10.78 ± 0.14	10.66 ± 0.13	10.57 ± 0.13	10.83 ± 0.13	10.66 ± 0.13
CMCC	10.64 ± 0.14	10.49 ± 0.12	10.78 ± 0.13	10.64 ± 0.14	10.80 ± 0.14	10.53 ± 0.13

Results are mean scores of 3 ± SE.

**Table 9:** Average carbohydrate content (%) of mushrooms harvested on different substrates.

Substrate type	Compost height					
	0.8 M			1.5 M		
	Composting period			Composting period		
	3 days	5 days	7 days	3 days	5 days	7 days
CM	74.61 ± 0.13	74.49 ± 0.14	74.68 ± 0.13	74.52 ± 0.12	74.61 ± 0.14	74.58 ± 0.13
NCM	74.80 ± 0.14	74.46 ± 0.12	73.55 ± 0.13	74.64 ± 0.14	73.34 ± 0.13	73.07 ± 0.13
NCMCC	73.64 ± 0.13	74.50 ± 0.13	74.80 ± 0.12	74.14 ± 013	74.72 ± 0.13	73.98 ± 0.12
CMCC	72.19 ± 0.13	73.88 ± 0.13	73.92 ± 0.14	74.51 ± 0.13	74.77 ± 0.14	74.80 ± 0.13

Results are mean scores of 3 ± SE.

**Table 10:** Effect of compost height, composting period, and substrate interactions on the physical characteristics of *Pleurotus ostreatus*.

Time	0.8 m compost height			1.5 m compost height	
	Substrate	Cap diameter	Stipe length	Cap diameter	Stipe length
3 days	cm	5.5^b^	5.5^c^	5.0^a^	4.1^a^
	ncm	3.6^a^	1.8^a^	5.1^a^	4.2^a^
	cmcc	5.5^b^	3.0^b^	5.4^b^	4.0^a^
	ncmcc	4.3^a^	3.4^b^	5.7^c^	5.2^c^

5 days	cm	5.6^b^	3.3^b^	5.6^c^	5.0^b^
	ncm	3.7^a^	1.8^a^	6.1^c^	5.1^c^
	cmcc	4.4^a^	3.0^b^	7.0^d^	5.0^b^
	ncmcc	5.6^b^	3.3^b^	6.1^c^	5.2^c^

7 days	cm	6.1^c^	4.0^b^	5.9^c^	4.9^b^
	ncm	4.0^a^	3.4^b^	6.0^c^	5.0^b^
	cmcc	6.5^c^	5.0^c^	6.6^d^	5.0^b^
	ncmcc	6.4^c^	5.2^c^	6.3^c^	5.1^c^

Means with the same letters in a column are not significantly different (*P* > 0.05).

Results are mean scores of 8.
